# Causal effect between gut microbiota and pancreatic cancer: a two-sample Mendelian randomization study

**DOI:** 10.1186/s12885-023-11493-y

**Published:** 2023-11-10

**Authors:** Zhichen Jiang, Yiping Mou, Huiju Wang, Li Li, Tianyu Jin, He Wang, Mingyang Liu, Weiwei Jin

**Affiliations:** 1 The Second School of Clinical Medicine, Zhejiang Chinese Medical University, Hangzhou, 310053 Zhejiang China; 2 Department of General Surgery, Devision of Dastroenterology and Pancreas, Affiliated People’s Hospital, Zhejiang Provincial People’s Hospital, Hangzhou Medical College, Hangzhou, 310014 Zhejiang China; 3 State Key Laboratory of Molecular Oncology, National Cancer Center, National Clinical Research Center for Cancer/Cancer Hospital, Chinese Academy of Medical Sciences and Peking Union Medical College, Beijing, 100021 China; 4 Key Laboratory of Gastroenterology of Zhejiang Province, Hangzhou, 310014 Zhejiang China

**Keywords:** Mendelian randomization study, Pancreatic cancer, Gut microbiota, FinnGen database, Causality

## Abstract

**Background:**

Gut microbiota (GM) comprises a vast and diverse community of microorganisms, and recent studies have highlighted the crucial regulatory roles of various GM and their secreted metabolites in pancreatic cancer (PC). However, the causal relationship between GM and PC has yet to be confirmed.

**Methods:**

In the present study, we used two-sample Mendelian randomization (MR) analysis to investigate the causal effect between GM and PC, with genome-wide association study (GWAS) from MiBioGen consortium as an exposure factor and PC GWAS data from FinnGen as an outcome factor. Inverse variance weighted (IVW) was used as the primary method for this study.

**Results:**

At the genus level, we observed that Senegalimassilia (OR: 0.635, 95% *CI*: 0.403–0.998, *P* = 0.049) exhibited a protective effect against PC, while Odoribacter (OR:1.899, 95%*CI*:1.157–3.116, *P* = 0.011), Ruminiclostridium 9(OR:1.976,95%*CI*:1.128–3.461, *P* = 0.017), Ruminococcaceae (UCG011)(OR:1.433, 95%CI:1.072–1.916, *P* = 0.015), and Streptococcus(OR:1.712, 95%CI:1.071–1.736, *P* = 0.025) were identified as causative factors for PC. Additionally, sensitivity analysis, Cochran’s Q test, the Mendelian randomization pleiotropy residual sum and outlier (MR-PRESSO), and MR-Egger regression indicated no heterogeneity, horizontal pleiotropy, or reverse causality between GM and PC.

**Conclusions:**

Our analysis establishes a causal effect between specific GM and PC, which may provide new insights into the potential pathogenic mechanisms of GM in PC and the assignment of effective therapeutic strategies.

**Supplementary Information:**

The online version contains supplementary material available at 10.1186/s12885-023-11493-y.

## Introduction

PC represents one of the most malignant gastrointestinal tumors, characterized by its stealthiness, aggressiveness, and high lethality, with a meager 5-year survival rate of only 11% [1]. In the United States, PC accounts for over 62,210 annual diagnoses and more than 49,830 deaths [1]. By 2040, PC is projected to surpass lung cancer as the second leading cause of cancer-related deaths in the US [2].

Current studies have revealed that the occurrence and progression of PC are associated with genetic mutations, dietary habits, and environmental factors [3, 4]. Among them, through the advancements in metagenomic and 16 S ribosomal RNA gene sequencing, researchers have discovered a correlation between GM and pancreatic diseases, including PC [5]. GM dysbiosis not only affects intestinal diseases directly [6, 7], but also extends its influence to extraintestinal organs, such as the pancreas and liver [8]. Studies have observed multiple changes in the microbiota of the oral cavity, gastrointestinal tract, and pancreas in PC patients compared to healthy individuals, highlighting the role of GM in PC [9]. This alteration in GM exerts its influence on PC through multiple mechanisms. Specifically, GM can interact with various risk factors for PC, such as obesity and diabetes, triggering an inflammatory response. This activation of the inflammatory response can facilitate the entry of GM and its secretions into the pancreas through different routes, including hematogenous spread, lymphatic metastasis, and pancreatic retrograde flow [10]. Furthermore, GM influences the development and progression of PC by modulating inflammatory responses, immune cell infiltration, and other mechanisms [11–13]. However, despite this, their causal effect has yet to be discovered.

MR Study is a statistical method widely used to analyze the causal relationship between exposure and outcome factors, which follows Mendel’s second law and relies on the independent random assignment of genetic variants during meiosis to realize a similar randomization effect as in Randomized Controlled Trials. Thus, MR can effectively overcome the confounders that occur in traditional studies and avoid reverse causality [14, 15]. To our knowledge, current MR-based studies have explored the causal relationships between GM and autoimmune diseases [16], colorectal cancer [17], and psychiatric disorders [18], but no MR-based studies have been performed to investigate the relationship between GM and PC.

In this study, we employed a two-sample MR approach to evaluate the causal effect between GM and PC, providing insights into the etiology and mechanisms of PC.

## Materials and methods

### Overall study design

**Fig. 1:**
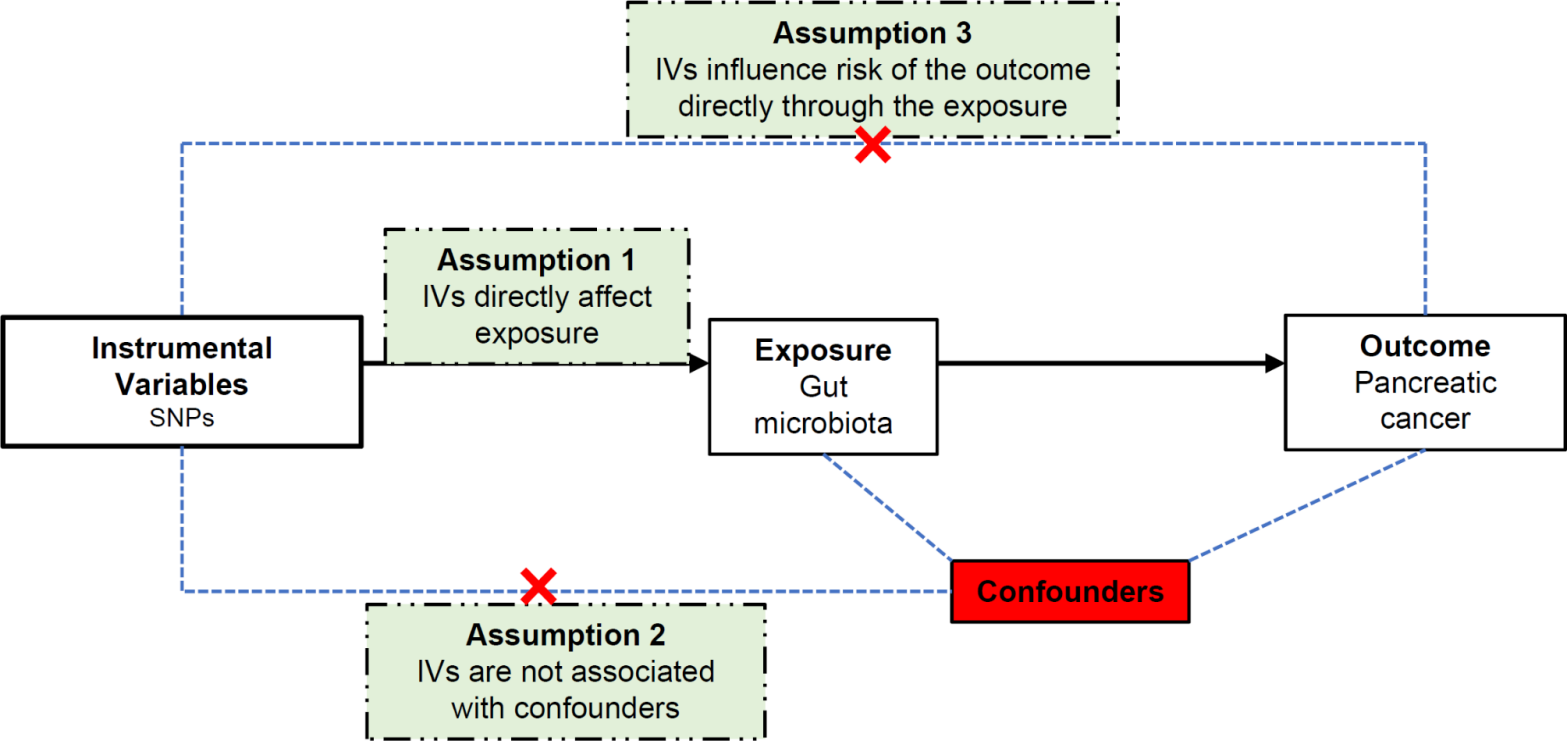
An overview of the study design. SNP: single nucleotide polymorphisms; IVs: instrumental variables

This study utilized pooled-level genetic data to conduct a two-sample MR analysis, aiming to investigate the causal effect between GM and PC. For this purpose, genetic variants that demonstrated significant associations with GM exposure were considered as instrumental variables (IVs), satisfying three crucial assumptions: the correlation assumption, independence assumption, and exclusion-limitation assumption (Fig. 1).

This study is based on publicly available abstract-level data from extensive genome-wide association study (GWAS) and consortia. Therefore, no additional ethical approval or consent to participate was required for this analysis.

### Data sources

In this study, the data were derived from extensive GWAS and publicly available GWAS data from consortia.

The GWAS summary statistics for GM were obtained from a MiBioGen consortium meta-analysis [19–23], This meta-analysis included a total of 18,340 individuals from 24 cohorts, with a majority of individuals having European ancestry (n = 13,266). The microbial composition was analyzed by targeting variable regions V4, V3-V4, and V1-V2 of the 16 S rRNA gene, and classification was performed using direct taxonomic sub boxes. At the genus level, 131 genera with an average abundance higher than 1% were identified, including 12 unknown genera. Therefore, a total of 119 genus-level taxonomic units were included in the analysis and analyzed separately.

The GWAS summary statistics for PC were obtained from the FinnGen Consortium R9 release and included 377,277 patients (210,870 females and 166,407 males) with 20,175,454 variables. This study used the “Malignant neoplasm of pancreas” phenotype and included 1416 cases and 287,137 controls after adjusting for age, sex, ten significant components, and genotyping cohort [24–26].

### Instrumental variables

The following selection criteria were used to select IVs: (1) At the beginning, the genome-wide significance threshold for single nucleotide polymorphisms (SNPs) associated with GM was set to *P* < 5 × 10^− 8^. Since the number of eligible IVs (*P* < 5 × 10^− 8^) was minimal, a relatively more comprehensive threshold ( *P* < 1.0 × 10^− 5^ ) was finally chosen [27], (2): IV1000 Genomes project European samples data were used as reference panel to calculate the linkage disequilibrium (LD) between SNPs, and the LD threshold was set to *r*^*2*^ < 0.001 with an aggregation window of 10,000 kb (clumping window size = 10,000 kb) [28], (3): remove SNPs with minor allele frequency (MAF) < 0.01, for MAF values no marked in the database, by querying the literature [27] as well as relevant databases(http://www.phenoscanner.medschl.cam.ac.uk/)0.4: If the specific requested SNP does not exist in the resulting GWAS, the SNP(proxy) located in the LD with the requested SNP(target) will be searched (*R*^*2*^ > 0.8). LD proxies were defined using 1000 genomes of the European sample data. To avoid strand orientation or distortion of allele coding, we removed palindromic SNPs [18]. 5: In order to exclude potential associations between IVs and risk factors for PC, we identified the risk factors for PC as: smoking, diabetes, alcohol consumption, and chronic pancreatitis, according to the NCCN guidelines [29]. And the IVs were analysed with these risk factors using the PhenoScanner database and excluded SNPs that were potentially associated with PC risk factors.

### F-statistic

The IVs included in the MR analysis should exhibit a significant association with the exposure. To assess the strength of the IVs, the F-statistic is commonly used. The F-statistic can be calculated using the formula: **F =** ***R***^***2***^**(n - k − 1) / k (1 -*R***^***2***^**)**, where ***R***^***2***^ is the proportion of the explained variance of the exposure by the genetic instrument, **n** is the sample size, and **k** denotes the number of IVs included. If the calculated F<10, it indicates a weak link between the IVs and the exposure, and such IVs were excluded from the analysis.

### MR

**Fig. 2:**
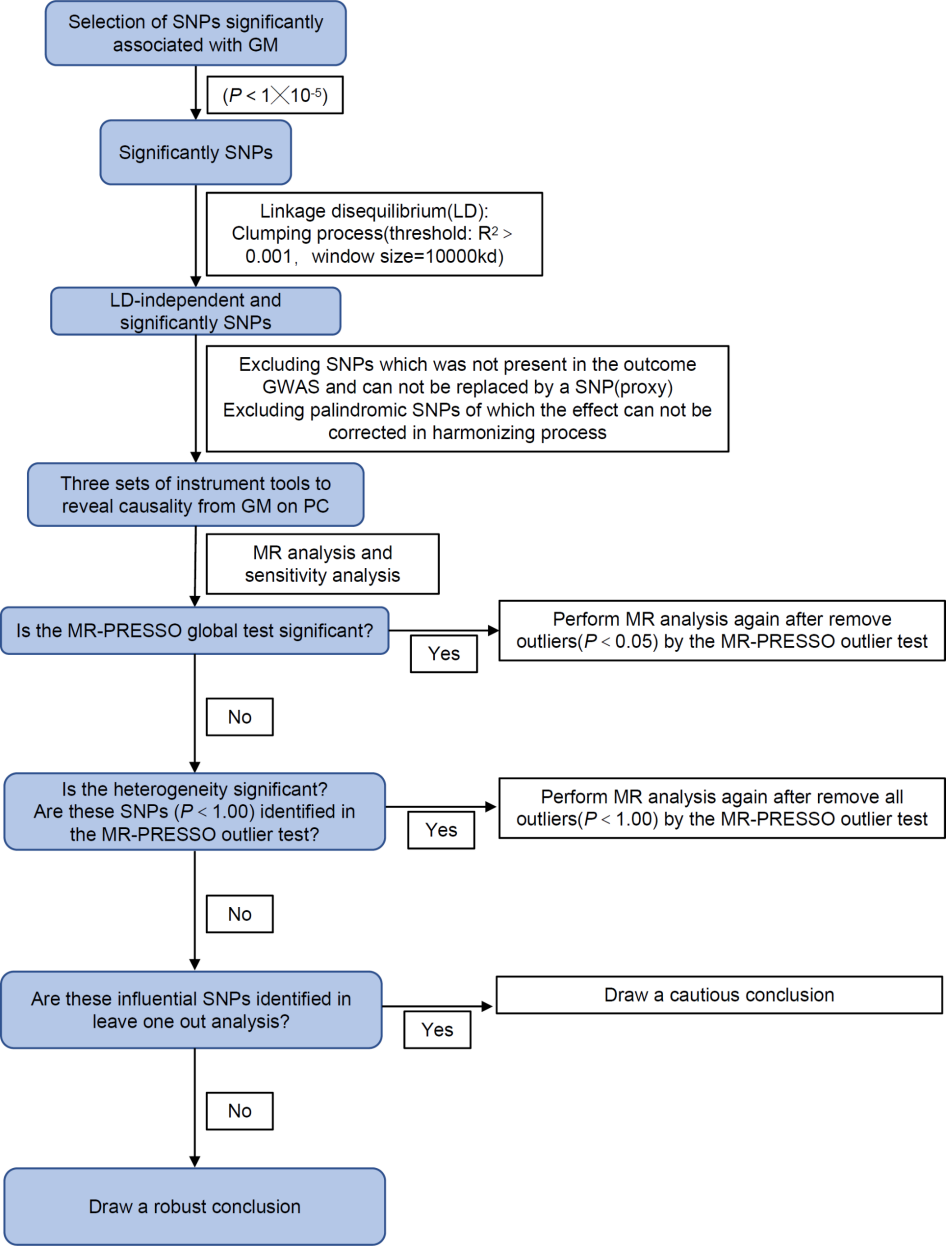
The flow chart of MR analysis. SNP: single nucleotide polymorphisms; MR: Mendelian Randomization

Our MR analysis was conducted following the guidelines outlined in the STROBE-MR statement [30](Table S1). The MR process flowchart is shown in Fig. 2.

To evaluate the causal estimates of GM on the risk of PC, we employed several MR methods, including inverse variance weighting (IVW) [31] the weighted median (WM) method [32], the MR-Egger test [33], Weighted mode (WMO) method, and robust adjusted profile score (RAPS) [34] method. The IVW method uses a meta-analysis method to combine Wald estimates of each SNP to obtain GM’s overall estimate of PC. If no horizontal pleiotropy is present, an unbiased result can be obtained by IVW linear regression [34, 35]. Therefore, in this study, IVW was employed as the primary method, while another four methods were used as complementary approaches.

Heterogeneity was assessed using Cochrane’s Q test, and IVs with *P* < 0.05 were considered heterogeneous. Additionally, the MR-Egger regression test was employed to examine the presence of horizontal pleiotropy in MR analysis. If *P* > 0.05, horizontal pleiotropy was considered not to be present. We would further analyze the pleiotropy using MR-PRESSO and remove possible outliers to ensure the accuracy of the results for GM taxa causally related to PC (based on IVW results), Furthermore, sensitivity analysis was conducted by iteratively removing each SNP to implement the leave-one-out method, aiming to verify the reliability and stability of the estimated causal effects [28].

### Statistical analysis

R software was used to conduct all statistical analyses (version 4.2.2). We performed MR of the causal link between GM and PC using the “TwoSample MR” package. 119 different MR Analyzes were conducted independently of each other, so we did not perform Bonferroni correction for multiple testing. *P* < 0.05 was considered statistically significant as evidence of a potential causal effect.

## Results

### Selection of instrumental variables

Based on the principles of instrumental variable selection, a total of 119 genus-level GMs containing 1198 SNPs (*P* < 1 × 10^5^) were finally identified as IVs in the MR analysis, and the details of all SNPs are detailed in Table S2.

### MR analysis

**Fig. 3:**
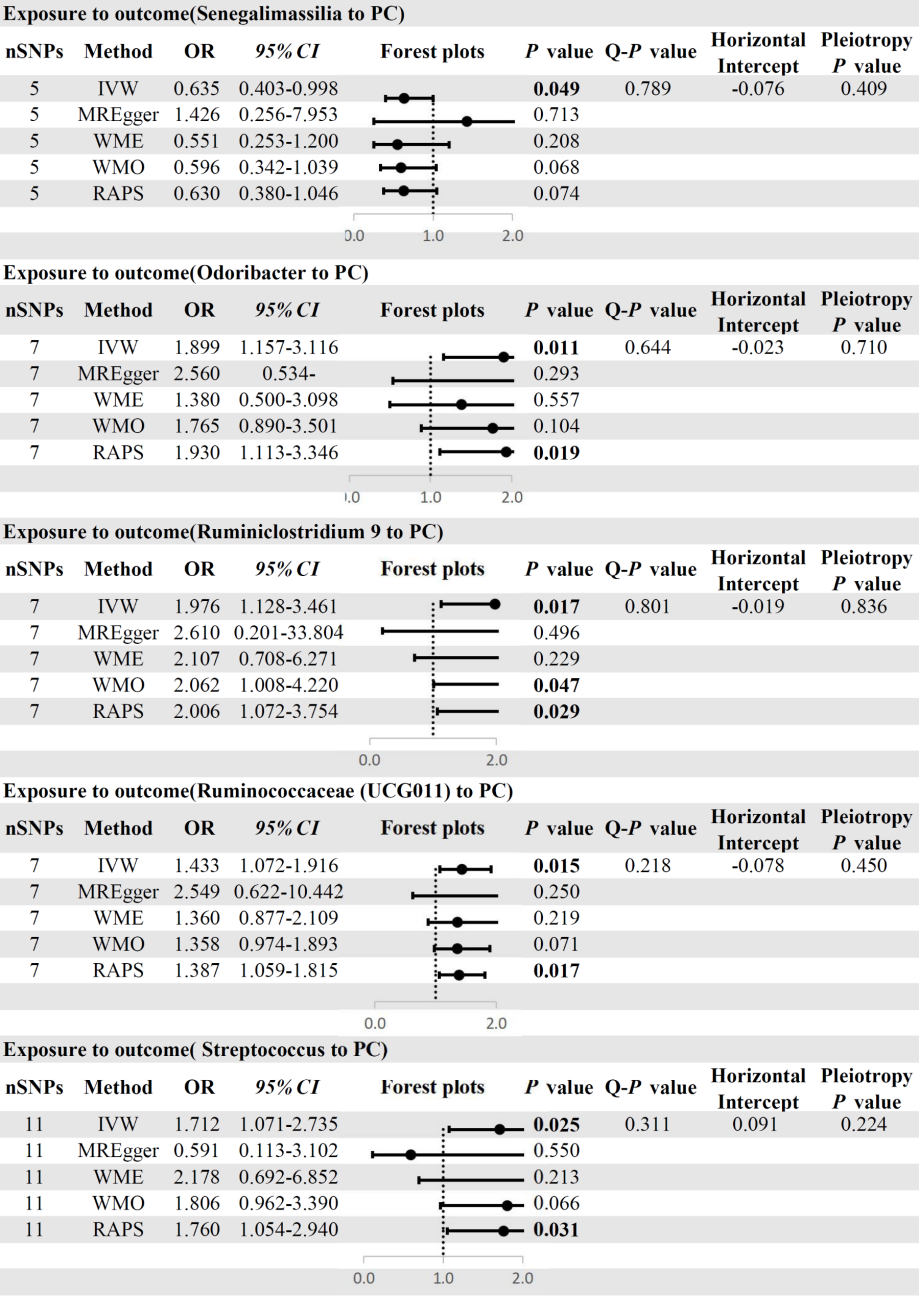
MR results of GM on PC. MR: Mendelian Randomization; GM: Gut microbiota; PC: Pancreatic cancer

IVW was chosen as the primary method for MR analysis because of its higher statistical efficacy. We identified six genus-level GMs (42 SNPs in total) that were causally associated with PC. Alloprevotella (OR: 0.752, 95% *CI*: 0.570–0.993, *P* = 0.045) was excluded because it was a weak instrumental variable (F = 9.8). We eventually identified five genus-level of GMs with the causal relationship with PC. Specifically, Senegalimassilia (OR: 0.635, 95% *CI*: 0.403–0.998, *P* = 0.049) was protective factors for PC. In contrast, Odoribacter (OR:1.899, 95%*CI*:1.157–3.116, *P* = 0.011), Ruminiclostridium 9(OR:1.976,95%*CI*:1.128–3.461, *P* = 0.017), Ruminococcaceae (UCG011)(OR:1.433, 95%CI:1.072–1.916, *P* = 0.015),and Streptococcus(OR:1.712, 95%CI:1.071–1.736, *P* = 0.025)were predisposing factors for PC(Fig. 3).

### Sensitivity analysis

**Fig. 4:**
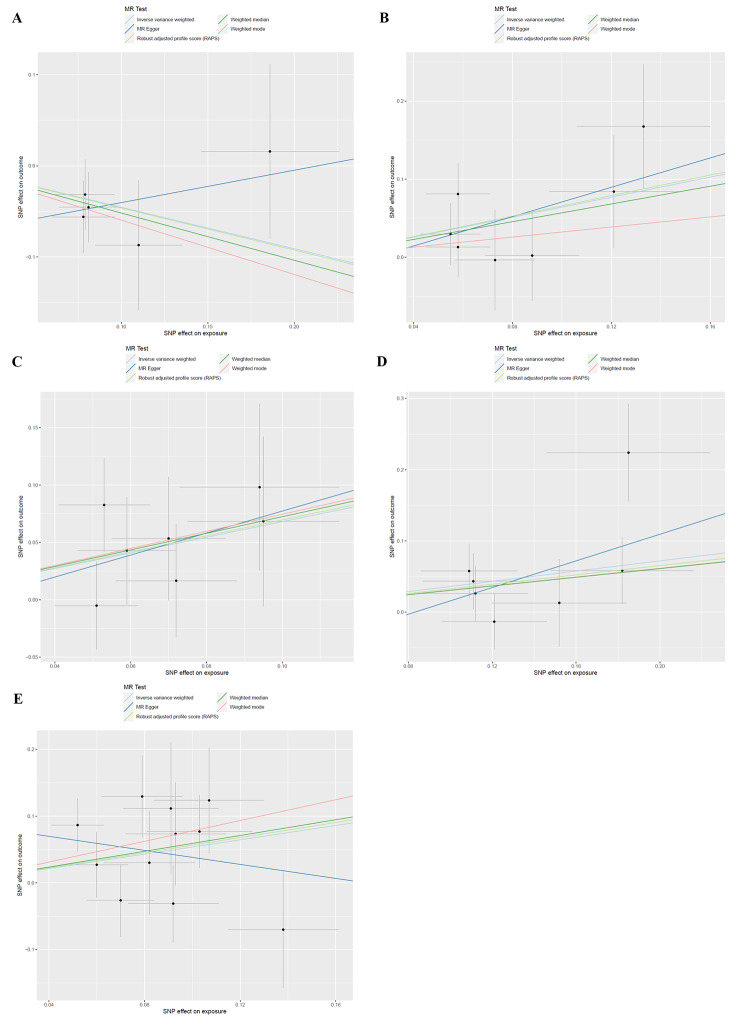
Scatter plots for MR analyses of the causal effect of GM on PC. A: Senegalimassilia;B: Odoribacter; C: Ruminiclostridium 9; D: Ruminococcaceae (UCG011);E: Streptococcus.SNP: single nucleotide polymorphisms; MR: Mendelian Randomization; PC: Pancreatic cancer

**Fig. 5:**
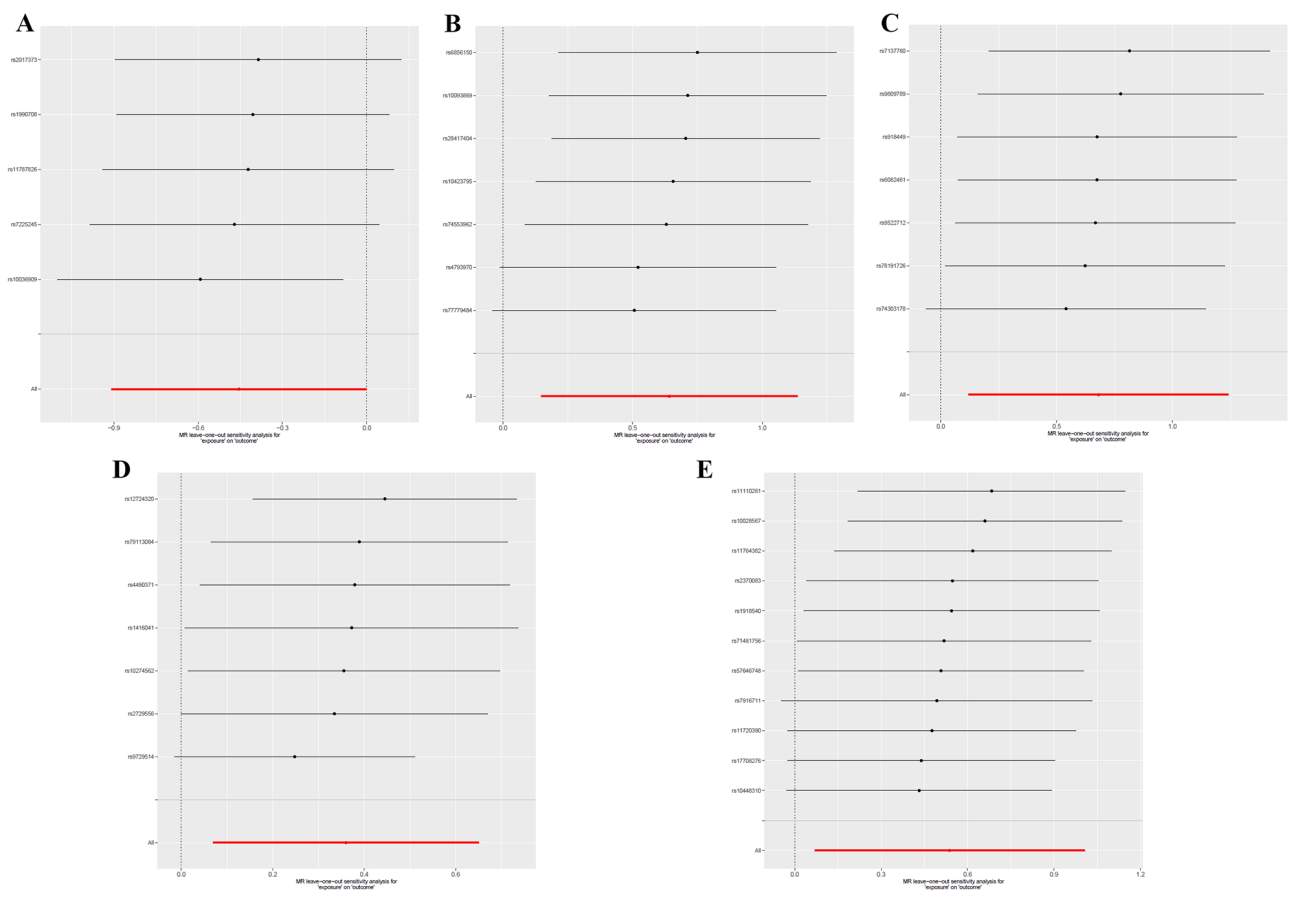
leave-one-out plots for MR analyses of the causal effect of GM on PC. A: Senegalimassilia;B: Odoribacter; C: Ruminiclostridium 9; D: Ruminococcaceae (UCG011);E: Streptococcus.SNP: single nucleotide polymorphisms; MR: Mendelian Randomization; PC: Pancreatic cancer

In these five causal effects, the F-statistic for IV was between 12.77 and 122.64 (Table S3), eliminating the bias for weak IV. Cochran’s Q test for IVW showed no significant heterogeneity for these IVs (Table S4), and MR-Egger regression intercept analysis found no horizontal pleiotropy (Table S5). Based on the Scatter plots (Fig. 4) and the leave-one-out plot (Fig. 5), we detected potential outliers for all five IVs, but further MR-PRESSO analysis did not reveal any significant outliers (Table S6). However, it is noteworthy that, in certain instances, the slope from the MR-Egger method was inverse to that observed in the IVW method. Although this did not reach statistical significance, it could hint at the presence of some form of pleiotropy. Despite our MR-Egger regression intercept analysis not revealing evidence of horizontal pleiotropy, this inverse relationship between the two methods should be considered, suggesting that potential pleiotropic effects might subtly influence our results.

## Discussions

In this study, we performed a MR analysis using the GWAS database of GM and PC to investigate the causal effect between them. Our findings revealed that at the genus level, Senegalimassilia was identified as protective factors, while Odoribacter, Ruminiclostridium 9, Ruminococcaceae (UCG011), and Streptococcus were associated with increased risk for PC.

The human’s gastrointestinal tract contains more than 1014 microorganisms and more than 5,000,000 genes, which can affect the normal physiology of the body by influencing metabolism as well as regulating the immune system, and many studies have discovered that dysbiosis of the GM is closely associated with diseases such as cancer, cardiovascular diseases, and psychiatric disorders [36]. While the pancreas was traditionally believed to be in a sterile environment due to its lack of direct contact with the intestine. However, recent studies have detected that pancreatic cancers can affect not only the type and abundance of GM but also the presence of GM can be detected in the pancreas of PC [37]. The exact mechanism of pancreatic flora formation is not precise. However, current studies have identified direct translocation through the pancreatic duct, metastasis through mesenteric lymph nodes, and hematogenous infection as potential routes of spread [38]. In PC, GM and its metabolites may cause chronic inflammation, while an unhealthy lifestyle can exacerbate this condition and thus induce tumorigenesis. In addition, abnormal GM can affect local intestinal immunity, T-cell development, and immune system maturation [10], all of which are contributing factors to the development and progression of PC.

The prognosis of PC is exceedingly poor, characterized by late-stage diagnosis, limited therapeutic efficacy, and high susceptibility to recurrence and metastasis (as cited in the literature). Addressing these challenges and finding ways to improve therapeutic efficacy, overcome treatment tolerance, identify high-risk groups, and discover appropriate biomarkers for PC has become paramount research directions for PC researchers. In this context, GM has emerged as a novel and promising avenue in PC research. Beyond its potential as a biomarker for PC, recent studies have highlighted various GM-based therapeutic approaches, such as probiotic therapy and fecal microbiota transplantation, as promising future directions [39]. Our study analyzed the GM associated with PC using an MR method based on publicly available GWAS data. Our findings revealed Senegalimassilia as a protective factor against PC, suggesting a promising direction for GM-based therapies. On the other hand, we identified Odoribacter, Ruminiclostridium 9, Ruminococcaceae (UC Ruminococcaceae (UCG011)), and Streptococcus as risk factors for PC, providing valuable guidance for the development of GM-based predictive models for PC.

About Senegalimassilia, it has been found to exhibit a higher abundance in cirrhotic patients with exacerbated steatosis and cirrhotic patients without extracellular fluid [40]. In addition, MR analyses suggest that Senegalimassilia may be a protective factor against hypertension [41]. These results indicate that Senegalimassilia may play a key role in regulating metabolic and inflammatory processes, which may also explain its role as a protective factor against PC. In contrast, Odoribacter is known for producing essential short-chain fatty acids and modulating intestinal barrier function and inflammatory processes, making it a promising therapeutic candidate for inflammatory bowel disease [42]. Yet its abundance was elevated in hepatocellular carcinoma [43], highlighting the divergent roles that a single GM may play in different diseases. Similar discoveries apply to Streptococcus, which exhibits increased abundance in various diseases, including esophageal cancer [44], colon cancer [45], and chronic pancreatitis [46], while other studies suggest a potential link between Streptococcus and longevity [47], implying diverse roles for Streptococcus in different diseases. Concerning Ruminiclostridium 9, it has demonstrated its regulatory effects on lipid metabolism, inflammation reduction, enhancement of intestinal barrier function, weight gain reduction, and improved insulin sensitivity in mice, effectively countering obesity development [48]. However, its specific role in pancreatic cancer remains unstudied, similar to Ruminococcaceae (UCG011), and both warrant further research in the context of pancreatic cancer.

Despite the valuable insights gained from this study, we acknowledge certain limitations. Firstly, the Mibiogen database, the largest multi-ethnic genome-wide meta-analysis of GM, includes samples from diverse populations, not exclusively composed of individuals of European origin. This heterogeneity may have impacted the reliability and generalizability of our conclusions. Secondly, the inherent limitations of the Mibiogen database compelled us to utilize pooled statistics from all subjects, restricting our capacity to conduct more specific subgroup analyses. Consequently, some potentially findings might have been obscured. Furthermore, the database constraints necessitated analyzing GM at the genus level rather than the strain level, possibly limiting the granularity of our results. Thirdly, due to the constraints of sequencing technology, the number of patients included in our study for each specific GM species was relatively small. This limited sample size and the scarcity of instrumental variables meeting the traditional GWAS significance thresholds (P < 5 × 10^− 8^) led us to use a significance threshold of (P < 1 × 10^− 5^) to obtain more comprehensive results. However, this adjustment may introduce some bias in the conclusions. Lastly, it is essential to acknowledge that the conclusions drawn from this study have not been externally validated in clinical settings, which represents a limitation that should be recognized.

## Conclusion

In conclusion, our two-sample Mendelian randomization (MR) study suggests a potential presence of a causal effect between GM and PC. Specifically, our findings revealed that at the genus level, Senegalimassilia was identified as protective factors, while Odoribacter, Ruminiclostridium 9, Ruminococcaceae (UCG011), and Streptococcus were associated with increased risk for PC. However, further original studies are needed to more comprehensively elucidate the underlying mechanisms that govern the relationship between GM and PC.

### Electronic supplementary material

Below is the link to the electronic supplementary material.

Supplementary Material 1

Supplementary Material 2

Supplementary Material 3

## Data Availability

The datasets analyzed during the current study are available in the MiBioGen repository(https://mibiogen.gcc.rug.nl/) [20] and the FinnGen repository (https://r9.finngen.fi/) [25, 26].
