# Supplementary material for: Causal effect between gut microbiota and pancreatic cancer: a two-sample Mendelian randomization study
Source: BMC Cancer. 2023 Nov 10;23:1091. doi: 10.1186/s12885-023-11493-y (PMC10636952; doi:10.1186/s12885-023-11493-y)
Supplement: Supplementary file 1 — Supplementary Material 1 [file 12885_2023_11493_MOESM1_ESM.docx]

**STROBE-MR checklist of recommended items to address in reports of Mendelian randomization studies**^1^ ^2^

| **Item No.** | **Section** | **Checklist item** | **Page No.** | **Relevant text from manuscript** |
| --- | --- | --- | --- | --- |
| 1 | **TITLE and ABSTRACT** | Indicate Mendelian randomization (MR) as the study’s design in the title and/or the abstract if that is a main purpose of the study | 1 | Causal effect between gut microbiota and pancreatic cancer: a Two-sample Mendelian Randomization Study |
|  | **INTRODUCTION** |  |  |  |
| 2 | **Background** | Explain the scientific background and rationale for the reported study. What is the exposure? Is a potential causal relationship between exposure and outcome plausible? Justify why MR is a helpful method to address the study question | 1-2 | researchers have discovered a correlation between GM and pancreatic diseases, including PC. GM dysbiosis not only affects intestinal diseases directly, but also extends its influence to extraintestinal organs, such as the pancreas and liver. Studies have observed multiple changes in the microbiota of the oral cavity, gastrointestinal tract, and pancreas in PC patients compared to healthy individuals, highlighting the role of GM in PC. |
| 3 | **Objectives** | State specific objectives clearly, including pre-specified causal hypotheses (if any). State that MR is a method that, under specific assumptions, intends to estimate causal effects | 2 | In this study, we employed a two-sample MR approach to evaluate the causal effect between GM and PC, providing insights into the etiology and mechanisms of PC. |
|  | **METHODS** |  |  |  |
| 4 | **Study design and data sources** | Present key elements of the study design early in the article. Consider including a table listing sources of data for all phases of the study. For each data source contributing to the analysis, describe the following: | 3 | In this study, the data were derived from extensive GWAS and publicly available GWAS data from consortia.  The GWAS summary statistics for GM were obtained from a MiBioGen consortium meta-analysis, This meta-analysis included a total of 18,340 individuals from 24 cohorts, with a majority of individuals having European ancestry (n=13,266). The microbial composition was analyzed by targeting variable regions V4, V3-V4, and V1-V2 of the 16S rRNA gene, and classification was performed using direct taxonomic sub boxes. At the genus level, 131 genera with an average abundance higher than 1% were identified, including 12 unknown genera. Therefore, a total of 119 genus-level taxonomic units were included in the analysis and analyzed separately.  The GWAS summary statistics for PC were obtained from the FinnGen Consortium R9 release and included 377277 patients (210870 females and 166407 males) with 20175454 variables. This study used the "Malignant neoplasm of pancreas" phenotype and included 1416 cases and 287137 controls after adjusting for age, sex, ten significant components, and genotyping cohort. |
|  | a) | Setting: Describe the study design and the underlying population, if possible. Describe the setting, locations, and relevant dates, including periods of recruitment, exposure, follow-up, and data collection, when available. | 3 | The GWAS summary statistics for GM were obtained from a MiBioGen consortium meta-analysis  The GWAS summary statistics for PC were obtained from the FinnGen Consortium R9 release and included 377277 patients (210870 females and 166407 males) with 20175454 variables. |
|  | b) | Participants: Give the eligibility criteria, and the sources and methods of selection of participants. Report the sample size, and whether any power or sample size calculations were carried out prior to the main analysis | 3 | The GWAS summary statistics for GM were obtained from a MiBioGen consortium meta-analysis[19-23], This meta-analysis included a total of 18,340 individuals from 24 cohorts, with a majority of individuals having European ancestry (n=13,266).  The GWAS summary statistics for PC were obtained from the FinnGen Consortium R9 release and included 377277 patients (210870 females and 166407 males) with 20175454 variables. This study used the "Malignant neoplasm of pancreas" phenotype and included 1416 cases and 287137 controls after adjusting for age, sex, ten significant components, and genotyping cohort |
|  | c) | Describe measurement, quality control and selection of genetic variants | 2 | This study utilized pooled-level genetic data to conduct a two-sample MR analysis, aiming to investigate the causal effect between GM and PC. For this purpose, genetic variants that demonstrated significant associations with GM exposure were considered as instrumental variables (IVs), satisfying three crucial assumptions: the correlation assumption, independence assumption, and exclusion-limitation assumption |
|  | d) | For each exposure, outcome, and other relevant variables, describe methods of assessment and diagnostic criteria for diseases | 3 | The microbial composition was analyzed by targeting variable regions V4, V3-V4, and V1-V2 of the 16S rRNA gene, and classification was performed using direct taxonomic sub boxes. At the genus level, 131 genera with an average abundance higher than 1% were identified, including 12 unknown genera  This study used the "Malignant neoplasm of pancreas" phenotype and included 1416 cases and 287137 controls after adjusting for age, sex, ten significant components, and genotyping cohort |
|  | e) | Provide details of ethics committee approval and participant informed consent, if relevant | 3 | This study is based on publicly available abstract-level data from extensive genome-wide association study (GWAS) and consortia. Therefore, no additional ethical approval or consent to participate was required for this analysis. |
| 5 | **Assumptions** | Explicitly state the three core IV assumptions for the main analysis (relevance, independence and exclusion restriction) as well assumptions for any additional or sensitivity analysis | 2 | This study utilized pooled-level genetic data to conduct a two-sample MR analysis, aiming to investigate the causal effect between GM and PC. For this purpose, genetic variants that demonstrated significant associations with GM exposure were considered as instrumental variables (IVs), satisfying three crucial assumptions: the correlation assumption, independence assumption, and exclusion-limitation assumption |
| 6 | **Statistical methods: main analysis** | Describe statistical methods and statistics used |  |  |
|  | a) | Describe how quantitative variables were handled in the analyses (i.e., scale, units, model) | 5 | To evaluate the causal estimates of GM on the risk of PC, we employed several MR methods, including inverse variance weighting (IVW) the weighted median (WM) method, the MR-Egger test, Weighted mode (WMO) method, and robust adjusted profile score (RAPS) method. |
|  | b) | Describe how genetic variants were handled in the analyses and, if applicable, how their weights were selected | 3-4 | (1) At the beginning, the genome-wide significance threshold for single nucleotide polymorphisms (SNPs) associated with GM was set to P < 5×10-8. Since the number of eligible IVs (P < 5×10-8) was minimal, a relatively more comprehensive threshold ( P < 1.0×10-5 ) was finally chosen[27], (2): IV1000 Genomes project European samples data were used as reference panel to calculate the linkage disequilibrium (LD) between SNPs, and the LD threshold was set to r2 < 0.001 with an aggregation window of 10,000 kb (clumping window size= 10,000 kb) [28], (3): remove SNPs with minor allele frequency (MAF) < 0.01, for MAF values no marked in the database, by querying the literature[27] as well as relevant databases(http://www.phenoscanner.medschl.cam.ac.uk/).4: If the specific requested SNP does not exist in the resulting GWAS, the SNP(proxy) located in the LD with the requested SNP(target) will be searched. LD proxies were defined using 1000 genomes of the European sample data. To avoid strand orientation or distortion of allele coding, we removed palindromic SNPs[18]. 5: In order to exclude potential associations between IVs and risk factors for PC, we identified the risk factors for PC as: smoking, diabetes, alcohol consumption, and chronic pancreatitis, according to the NCCN guidelines[29]. And the IVs were analysed with these risk factors using the PhenoScanner database and excluded SNPs that were potentially associated with PC risk factors. |
|  | c) | Describe the MR estimator (e.g. two-stage least squares, Wald ratio) and related statistics. Detail the included covariates and, in case of two-sample MR, whether the same covariate set was used for adjustment in the two samples | 3 | This study used the "Malignant neoplasm of pancreas" phenotype and included 1416 cases and 287137 controls after adjusting for age, sex, ten significant components, and genotyping cohort |
|  | d) | Explain how missing data were addressed | 3-4 | (1) At the beginning, the genome-wide significance threshold for single nucleotide polymorphisms (SNPs) associated with GM was set to P < 5×10-8. Since the number of eligible IVs (P < 5×10-8) was minimal, a relatively more comprehensive threshold ( P < 1.0×10-5 ) was finally chosen[27], (2): IV1000 Genomes project European samples data were used as reference panel to calculate the linkage disequilibrium (LD) between SNPs, and the LD threshold was set to r2 < 0.001 with an aggregation window of 10,000 kb (clumping window size= 10,000 kb) [28], (3): remove SNPs with minor allele frequency (MAF) < 0.01, for MAF values no marked in the database, by querying the literature[27] as well as relevant databases(http://www.phenoscanner.medschl.cam.ac.uk/).4: If the specific requested SNP does not exist in the resulting GWAS, the SNP(proxy) located in the LD with the requested SNP(target) will be searched. LD proxies were defined using 1000 genomes of the European sample data. To avoid strand orientation or distortion of allele coding, we removed palindromic SNPs[18]. 5: In order to exclude potential associations between IVs and risk factors for PC, we identified the risk factors for PC as: smoking, diabetes, alcohol consumption, and chronic pancreatitis, according to the NCCN guidelines[29]. And the IVs were analysed with these risk factors using the PhenoScanner database and excluded SNPs that were potentially associated with PC risk factors. |
|  | e) | If applicable, indicate how multiple testing was addressed | NA |  |
| 7 | **Assessment of assumptions** | Describe any methods or prior knowledge used to assess the assumptions or justify their validity | 6 | Heterogeneity was assessed using Cochrane's Q test, and IVs with P<0.05 were considered heterogeneous. Additionally, the MR-Egger regression test was employed to examine the presence of horizontal pleiotropy in MR analysis. If P > 0.05, horizontal pleiotropy was considered not to be present. We would further analyze the pleiotropy using MR-PRESSO and remove possible outliers to ensure the accuracy of the results for GM taxa causally related to PC (based on IVW results), Furthermore, sensitivity analysis was conducted by iteratively removing each SNP to implement the leave-one-out method, aiming to verify the reliability and stability of the estimated causal effects |
| 8 | **Sensitivity analyses and additional analyses** | Describe any sensitivity analyses or additional analyses performed (e.g. comparison of effect estimates from different approaches, independent replication, bias analytic techniques, validation of instruments, simulations) | 6 | Heterogeneity was assessed using Cochrane's Q test, and IVs with P<0.05 were considered heterogeneous. Additionally, the MR-Egger regression test was employed to examine the presence of horizontal pleiotropy in MR analysis. If P > 0.05, horizontal pleiotropy was considered not to be present. We would further analyze the pleiotropy using MR-PRESSO and remove possible outliers to ensure the accuracy of the results for GM taxa causally related to PC (based on IVW results), Furthermore, sensitivity analysis was conducted by iteratively removing each SNP to implement the leave-one-out method, aiming to verify the reliability and stability of the estimated causal effects. |
| 9 | **Software and pre-registration** |  |  |  |
|  | a) | Name statistical software and package(s), including version and settings used | 6 | R software was used to conduct all statistical analyses (version 4.2.2). We performed MR of the causal link between GM and PC using the "TwoSample MR" package. P < 0.05 was considered statistically significant as evidence of a potential causal effect. |
|  | b) | State whether the study protocol and details were pre-registered (as well as when and where) | 6 | R software was used to conduct all statistical analyses (version 4.2.2). We performed MR of the causal link between GM and PC using the "TwoSample MR" package. P < 0.05 was considered statistically significant as evidence of a potential causal effect. |
|  | **RESULTS** |  |  |  |
| 10 | **Descriptive data** |  |  |  |
|  | a) | Report the numbers of individuals at each stage of included studies and reasons for exclusion. Consider use of a flow diagram | 5 | Our MR analysis was conducted following the guidelines outlined in the STROBE-MR statement(Supple Table1). The MR process flowchart is shown in Fig 2 |
|  | b) | Report summary statistics for phenotypic exposure(s), outcome(s), and other relevant variables (e.g. means, SDs, proportions) | 6 | the details of all SNPs are detailed in Table S2 |
|  | c) | If the data sources include meta-analyses of previous studies, provide the assessments of heterogeneity across these studies | NA |  |
|  | d) | For two-sample MR:  i.  Provide justification of the similarity of the genetic variant-exposure associations between the exposure and outcome samples  ii.  Provide information on the number of individuals who overlap between the exposure and outcome studies | 3-4 | The following selection criteria were used to select IVs: (1) At the beginning, the genome-wide significance threshold for single nucleotide polymorphisms (SNPs) associated with GM was set to P < 5×10-8. Since the number of eligible IVs (P < 5×10-8) was minimal, a relatively more comprehensive threshold ( P < 1.0×10-5 ) was finally chosen, (2): IV1000 Genomes project European samples data were used as reference panel to calculate the linkage disequilibrium (LD) between SNPs, and the LD threshold was set to r2 < 0.001 with an aggregation window of 10,000 kb (clumping window size= 10,000 kb) , (3): remove SNPs with minor allele frequency (MAF) < 0.01, for MAF values no marked in the database, by querying the literature as well as relevant databases(http://www.phenoscanner.medschl.cam.ac.uk/).4: To avoid strand orientation or distortion of allele coding, we removed palindromic SNPs. 5: In order to exclude potential associations between IVs and risk factors for PC, we identified the risk factors for PC as: smoking, diabetes, alcohol consumption, and chronic pancreatitis, according to the NCCN guidelines. And the IVs were analysed with these risk factors using the PhenoScanner database and excluded SNPs that were potentially associated with PC risk factors. |
| 11 | **Main results** |  |  |  |
|  | a) | Report the associations between genetic variant and exposure, and between genetic variant and outcome, preferably on an interpretable scale | 6 | Based on the principles of instrumental variable selection, a total of 119 genus-level GMs containing 1198 SNPs (P < 1×105) were finally identified as IVs in the MR analysis, and the details of all SNPs are detailed in Table S2. |
|  | b) | Report MR estimates of the relationship between exposure and outcome, and the measures of uncertainty from the MR analysis, on an interpretable scale, such as odds ratio or relative risk per SD difference | 6 | Based on the principles of instrumental variable selection, a total of 119 genus-level GMs containing 1198 SNPs (P < 1×105) were finally identified as IVs in the MR analysis, and the details of all SNPs are detailed in Table S2. |
|  | c) | If relevant, consider translating estimates of relative risk into absolute risk for a meaningful time period | NA |  |
|  | d) | Consider plots to visualize results (e.g. forest plot, scatterplot of associations between genetic variants and outcome versus between genetic variants and exposure) | 6 | IVW was chosen as the primary method for MR analysis because of its higher statistical efficacy. We identified six genus-level GMs (42 SNPs in total) that were causally associated with PC. Alloprevotella (OR: 0.752, 95% CI: 0.570-0.993, P=0.045) was excluded because it was a weak instrumental variable (F=9.8). We eventually identified five genus-level of GMs with the causal relationship with PC. Specifically, Senegalimassilia (OR: 0.635, 95% CI: 0.403-0.998, P=0.049) was protective factors for PC. In contrast, Odoribacter (OR:1.899, 95%CI:1.157-3.116, P=0.011), Ruminiclostridium 9(OR:1.976,95%CI:1.128-3.461, P=0.017), Ruminococcaceae (UCG011)(OR:1.433, 95%CI:1.072-1.916, P=0.015),and Streptococcus(OR:1.712, 95%CI:1.071-1.736, P=0.025)were predisposing factors for PC(Fig 3) |
| 12 | **Assessment of assumptions** |  |  |  |
|  | a) | Report the assessment of the validity of the assumptions | 6 | IVW was chosen as the primary method for MR analysis because of its higher statistical efficacy. We identified six genus-level GMs (42 SNPs in total) that were causally associated with PC. Alloprevotella (OR: 0.752, 95% CI: 0.570-0.993, P=0.045) was excluded because it was a weak instrumental variable (F=9.8). We eventually identified five genus-level of GMs with the causal relationship with PC. Specifically, Senegalimassilia (OR: 0.635, 95% CI: 0.403-0.998, P=0.049) was protective factors for PC. In contrast, Odoribacter (OR:1.899, 95%CI:1.157-3.116, P=0.011), Ruminiclostridium 9(OR:1.976,95%CI:1.128-3.461, P=0.017), Ruminococcaceae (UCG011)(OR:1.433, 95%CI:1.072-1.916, P=0.015),and Streptococcus(OR:1.712, 95%CI:1.071-1.736, P=0.025)were predisposing factors for PC(Fig 3) |
|  | b) | Report any additional statistics (e.g., assessments of heterogeneity across genetic variants, such as *I^2^*, Q statistic or E-value) | 6 | IVW was chosen as the primary method for MR analysis because of its higher statistical efficacy. We identified six genus-level GMs (42 SNPs in total) that were causally associated with PC. Alloprevotella (OR: 0.752, 95% CI: 0.570-0.993, P=0.045) was excluded because it was a weak instrumental variable (F=9.8). We eventually identified five genus-level of GMs with the causal relationship with PC. Specifically, Senegalimassilia (OR: 0.635, 95% CI: 0.403-0.998, P=0.049) was protective factors for PC. In contrast, Odoribacter (OR:1.899, 95%CI:1.157-3.116, P=0.011), Ruminiclostridium 9(OR:1.976,95%CI:1.128-3.461, P=0.017), Ruminococcaceae (UCG011)(OR:1.433, 95%CI:1.072-1.916, P=0.015),and Streptococcus(OR:1.712, 95%CI:1.071-1.736, P=0.025)were predisposing factors for PC(Fig 3) |
| 13 | **Sensitivity analyses and additional analyses** |  |  |  |
|  | a) | Report any sensitivity analyses to assess the robustness of the main results to violations of the assumptions | 7-8 | In these five causal effects, the F-statistic for IV was between 12.77 and 122.64 (Table S3), eliminating the bias for weak IV. Cochran's Q test for IVW showed no significant heterogeneity for these IVs (Table S4), and MR-Egger regression intercept analysis found no horizontal pleiotropy (TableS5). Based on the Scatter plots (FigS1) and the leave-one-out plot (Fig 4), we detected potential outliers for all five IVs, but further MR-PRESSO analysis did not reveal any significant outliers (TableS6). Therefore, there is insufficient evidence for horizontal pleiotropy between these GMs and PC. |
|  | b) | Report results from other sensitivity analyses or additional analyses | 7-8 | In these five causal effects, the F-statistic for IV was between 12.77 and 122.64 (Table S3), eliminating the bias for weak IV. Cochran's Q test for IVW showed no significant heterogeneity for these IVs (Table S4), and MR-Egger regression intercept analysis found no horizontal pleiotropy (TableS5). Based on the Scatter plots (FigS1) and the leave-one-out plot (Fig 4), we detected potential outliers for all five IVs, but further MR-PRESSO analysis did not reveal any significant outliers (TableS6). Therefore, there is insufficient evidence for horizontal pleiotropy between these GMs and PC. |
|  | c) | Report any assessment of direction of causal relationship (e.g., bidirectional MR) | 7-8 | In these five causal effects, the F-statistic for IV was between 12.77 and 122.64 (Table S3), eliminating the bias for weak IV. Cochran's Q test for IVW showed no significant heterogeneity for these IVs (Table S4), and MR-Egger regression intercept analysis found no horizontal pleiotropy (TableS5). Based on the Scatter plots (FigS1) and the leave-one-out plot (Fig 4), we detected potential outliers for all five IVs, but further MR-PRESSO analysis did not reveal any significant outliers (TableS6). Therefore, there is insufficient evidence for horizontal pleiotropy between these GMs and PC. |
|  | d) | When relevant, report and compare with estimates from non-MR analyses | 7-8 | In these five causal effects, the F-statistic for IV was between 12.77 and 122.64 (Table S3), eliminating the bias for weak IV. Cochran's Q test for IVW showed no significant heterogeneity for these IVs (Table S4), and MR-Egger regression intercept analysis found no horizontal pleiotropy (TableS5). Based on the Scatter plots (FigS1) and the leave-one-out plot (Fig 4), we detected potential outliers for all five IVs, but further MR-PRESSO analysis did not reveal any significant outliers (TableS6). Therefore, there is insufficient evidence for horizontal pleiotropy between these GMs and PC. |
|  | e) | Consider additional plots to visualize results (e.g., leave-one-out analyses) | 7-8 | In these five causal effects, the F-statistic for IV was between 12.77 and 122.64 (Table S3), eliminating the bias for weak IV. Cochran's Q test for IVW showed no significant heterogeneity for these IVs (Table S4), and MR-Egger regression intercept analysis found no horizontal pleiotropy (TableS5). Based on the Scatter plots (FigS1) and the leave-one-out plot (Fig 4), we detected potential outliers for all five IVs, but further MR-PRESSO analysis did not reveal any significant outliers (TableS6). Therefore, there is insufficient evidence for horizontal pleiotropy between these GMs and PC. |
|  | **DISCUSSION** |  |  |  |
| 14 | **Key results** | Summarize key results with reference to study objectives | 9 | In this study, we performed a MR analysis using the GWAS database of GM and PC to investigate the causal effect between them. Our findings revealed that at the genus level, Senegalimassilia was identified as protective factors, while Odoribacter, Ruminiclostridium 9, Ruminococcaceae (UCG011), and Streptococcus were associated with increased risk for PC. |
| 15 | **Limitations** | Discuss limitations of the study, taking into account the validity of the IV assumptions, other sources of potential bias, and imprecision. Discuss both direction and magnitude of any potential bias and any efforts to address them | 11 | Despite the valuable insights gained from this study, we acknowledge certain limitations. Firstly, the Mibiogen database, the largest multi-ethnic genome-wide meta-analysis of GM, includes samples from diverse populations, not exclusively composed of individuals of European origin. This heterogeneity may have impacted the reliability and generalizability of our conclusions. Secondly, the inherent limitations of the Mibiogen database compelled us to utilize pooled statistics from all subjects, restricting our capacity to conduct more specific subgroup analyses. Consequently, some potentially findings might have been obscured. Furthermore, the database constraints necessitated analyzing GM at the genus level rather than the strain level, possibly limiting the granularity of our results. Thirdly, due to the constraints of sequencing technology, the number of patients included in our study for each specific GM species was relatively small. This limited sample size and the scarcity of instrumental variables meeting the traditional GWAS significance thresholds (P<5×10-8) led us to use a significance threshold of (P<1×10-5) to obtain more comprehensive results. However, this adjustment may introduce some bias in the conclusions. Lastly, it is essential to acknowledge that the conclusions drawn from this study have not been externally validated in clinical settings, which represents a limitation that should be recognized. |
| 16 | **Interpretation** |  |  |  |
|  | a) | Meaning: Give a cautious overall interpretation of results in the context of their limitations and in comparison with other studies | 11 | Despite the valuable insights gained from this study, we acknowledge certain limitations. Firstly, the Mibiogen database, the largest multi-ethnic genome-wide meta-analysis of GM, includes samples from diverse populations, not exclusively composed of individuals of European origin. This heterogeneity may have impacted the reliability and generalizability of our conclusions. Secondly, the inherent limitations of the Mibiogen database compelled us to utilize pooled statistics from all subjects, restricting our capacity to conduct more specific subgroup analyses. Consequently, some potentially findings might have been obscured. Furthermore, the database constraints necessitated analyzing GM at the genus level rather than the strain level, possibly limiting the granularity of our results. Thirdly, due to the constraints of sequencing technology, the number of patients included in our study for each specific GM species was relatively small. This limited sample size and the scarcity of instrumental variables meeting the traditional GWAS significance thresholds (P<5×10-8) led us to use a significance threshold of (P<1×10-5) to obtain more comprehensive results. However, this adjustment may introduce some bias in the conclusions. Lastly, it is essential to acknowledge that the conclusions drawn from this study have not been externally validated in clinical settings, which represents a limitation that should be recognized. |
|  | b) | Mechanism: Discuss underlying biological mechanisms that could drive a potential causal relationship between the investigated exposure and the outcome, and whether the gene-environment equivalence assumption is reasonable. Use causal language carefully, clarifying that IV estimates may provide causal effects only under certain assumptions | 9-10 | The human's gastrointestinal tract contains more than 1014 microorganisms and more than 5,000,000 genes, which can affect the normal physiology of the body by influencing metabolism as well as regulating the immune system, and many studies have discovered that dysbiosis of the GM is closely associated with diseases such as cancer, cardiovascular diseases, and psychiatric disorders[36]. While the pancreas was traditionally believed to be in a sterile environment due to its lack of direct contact with the intestine. However, recent studies have detected that pancreatic cancers can affect not only the type and abundance of GM but also the presence of GM can be detected in the pancreas of PC[37]. The exact mechanism of pancreatic flora formation is not precise. However, current studies have identified direct translocation through the pancreatic duct, metastasis through mesenteric lymph nodes, and hematogenous infection as potential routes of spread[38]. In PC, GM and its metabolites may cause chronic inflammation, while an unhealthy lifestyle can exacerbate this condition and thus induce tumorigenesis. In addition, abnormal GM can affect local intestinal immunity, T-cell development, and immune system maturation[10], all of which are contributing factors to the development and progression of PC. |
|  | c) | Clinical relevance: Discuss whether the results have clinical or public policy relevance, and to what extent they inform effect sizes of possible interventions | 10 | Our findings revealed Senegalimassilia as a protective factor against PC, suggesting a promising direction for GM-based therapies. On the other hand, we identified Odoribacter, Ruminiclostridium 9, Ruminococcaceae (UC Ruminococcaceae (UCG011)), and Streptococcus as risk factors for PC, providing valuable guidance for the development of GM-based predictive models for PC. |
| 17 | **Generalizability** | Discuss the generalizability of the study results (a) to other populations, (b) across other exposure periods/timings, and (c) across other levels of exposure | 10-11 | Despite the valuable insights gained from this study, we acknowledge certain limitations. Firstly, the Mibiogen database, the largest multi-ethnic genome-wide meta-analysis of GM, includes samples from diverse populations, not exclusively composed of individuals of European origin. This heterogeneity may have impacted the reliability and generalizability of our conclusions. Secondly, the inherent limitations of the Mibiogen database compelled us to utilize pooled statistics from all subjects, restricting our capacity to conduct more specific subgroup analyses. Consequently, some potentially findings might have been obscured. Furthermore, the database constraints necessitated analyzing GM at the genus level rather than the strain level, possibly limiting the granularity of our results. Thirdly, due to the constraints of sequencing technology, the number of patients included in our study for each specific GM species was relatively small. This limited sample size and the scarcity of instrumental variables meeting the traditional GWAS significance thresholds (P<5×10-8) led us to use a significance threshold of (P<1×10-5) to obtain more comprehensive results. However, this adjustment may introduce some bias in the conclusions. Lastly, it is essential to acknowledge that the conclusions drawn from this study have not been externally validated in clinical settings, which represents a limitation that should be recognized. |
|  | **OTHER INFORMATION** |  |  |  |
| 18 | **Funding** | Describe sources of funding and the role of funders in the present study and, if applicable, sources of funding for the databases and original study or studies on which the present study is based | 11 | This study was supported by Scientific research fund of national health commision of China, Key health science and technology program of Zhejiang Province(WKJ-ZJ-2201) and Key Project of social welfare program of Zhejiang Science and Technology Department,’Lingyan’Program(2022C03099) |
| 19 | **Data and data sharing** | Provide the data used to perform all analyses or report where and how the data can be accessed, and reference these sources in the article. Provide the statistical code needed to reproduce the results in the article, or report whether the code is publicly accessible and if so, where | 11 | The datasets analyzed during the current study are available in the MiBioGen repository(<https://mibiogen.gcc.rug.nl/>)[20] and the FinnGen repository (https://r9.finngen.fi/)[25, 26]. |
| 20 | **Conflicts of Interest** | All authors should declare all potential conflicts of interest | 11 | The authors declare that they have no conflict of interest. |

This checklist is copyrighted by the Equator Network under the Creative Commons Attribution 3.0 Unported (CC BY 3.0) license.

1. Skrivankova VW, Richmond RC, Woolf BAR, Yarmolinsky J, Davies NM, Swanson SA, et al. Strengthening the Reporting of Observational Studies in Epidemiology using Mendelian Randomization (STROBE-MR) Statement. JAMA. 2021;under review.

2. Skrivankova VW, Richmond RC, Woolf BAR, Davies NM, Swanson SA, VanderWeele TJ, et al. Strengthening the Reporting of Observational Studies in Epidemiology using Mendelian Randomisation (STROBE-MR): Explanation and Elaboration. BMJ. 2021;375:n2233.
