# Supplementary material for: Causal effect between gut microbiota and pancreatic cancer: a two-sample Mendelian randomization study
Source: BMC Cancer. 2023 Nov 10;23:1091. doi: 10.1186/s12885-023-11493-y (PMC10636952; doi:10.1186/s12885-023-11493-y)
Supplement: Supplementary file 3 — Supplementary Material 3 [file 12885_2023_11493_MOESM3_ESM.docx]

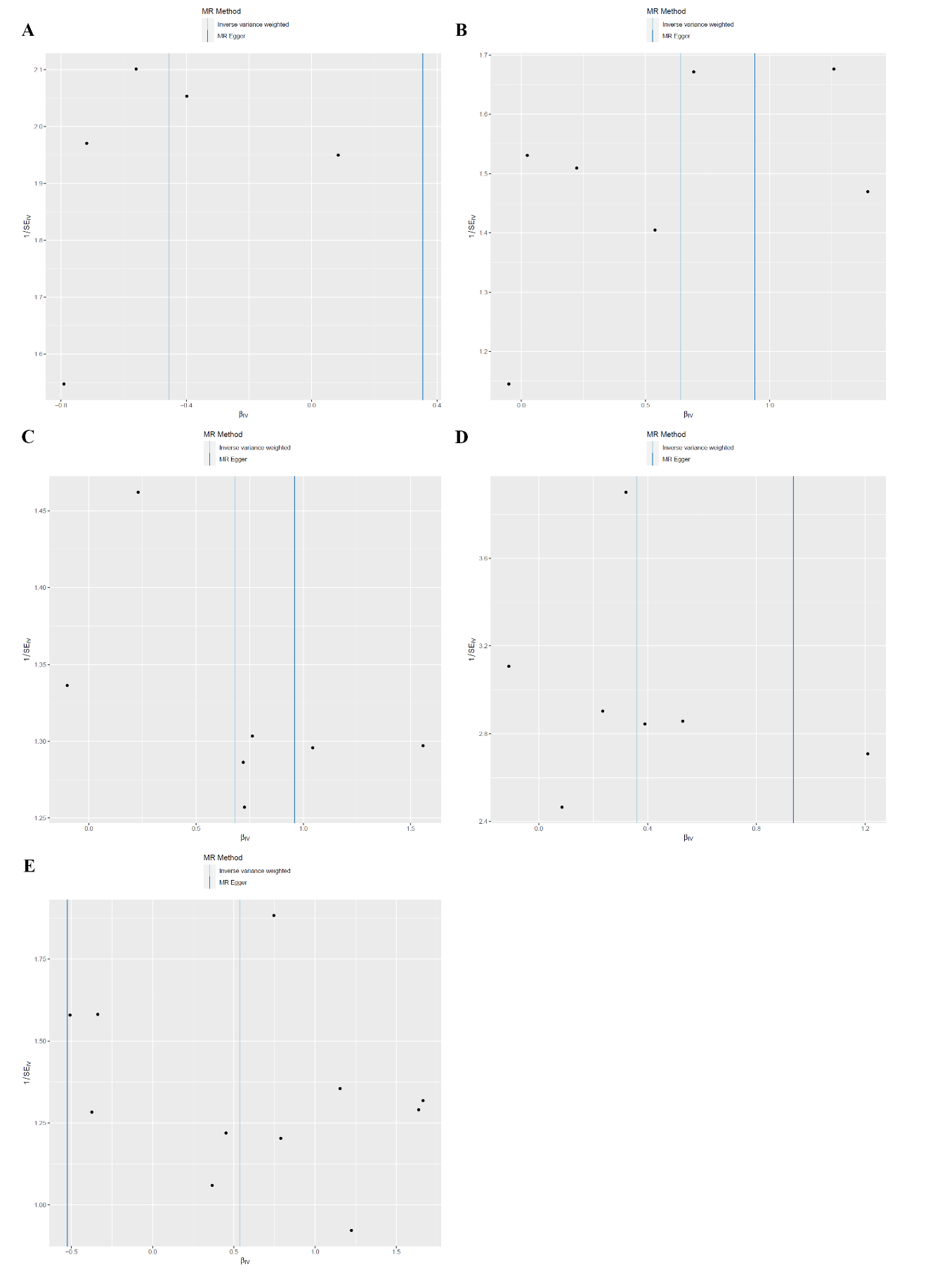


FigS1: Scatter plot for MR analyses of the causal effect of GM on PC. A: Senegalimassilia;B: Odoribacter; C: Ruminiclostridium 9; D: Ruminococcaceae (UCG011);E: Streptococcus.SNP: single nucleotide polymorphisms; MR: Mendelian Randomization; PC: Pancreatic cancer
